# Distal chromosome 1q aberrations and initial response to ibrutinib in central nervous system relapsed mantle cell lymphoma

**DOI:** 10.1016/j.lrr.2021.100255

**Published:** 2021-06-01

**Authors:** Marcus Høy Hansen, Karen Juul-Jensen, Oriane Cédile, Stephanie Kavan, Michael Boe Møller, Jacob Haaber, Charlotte Guldborg Nyvold

**Affiliations:** a Haematology-Pathology Research Laboratory, Research Unit for Haematology and Research Unit for Pathology, University of Southern Denmark and Odense University Hospital, Odense, Denmark; b Department of Clinical Genetics, Odense University Hospital, Denmark

**Keywords:** Mantle cell lymphoma, Central nervous system relapse, Copy number alteration, Chromosome 1 aberration, Ibrutinib response

## Abstract

Relapse involving the central nervous system (CNS) is an infrequent event in the progression of mantle cell lymphoma (MCL) with an incidence of approximately four percent. We report four cases of MCL with CNS relapse. In three of the four patients a large chromosomal copy-number alteration (CNA) of 1q was demonstrated together with TP53 mutation/deletion. These patients experienced brief response to ibrutinib, whereas a fourth patient harboring mutated ATM demonstrated a long-term effect to ibrutinib and no CNA. Although it is unclear whether chromosome 1q CNA contribute to specific phenotypes these reports may be of value as such lesions are uncommon features of MCL.

## 1 Introduction

Infiltration of the central nervous system (CNS) is an infrequent event in the progression of mantle cell lymphoma (MCL) and is estimated to occur in 4% of patients with primary systemic disease. [2, 3]. Taking into account the relative rarity of this malignancy, which is generally reported to be less than 10% of the non-Hodgkin's lymphomas, it is difficult to shed light on specific molecular lesions, if any, leading to the progression of CNS involvement. The severity of CNS progression, with the survival of only a few months, emphasizes the importance of further investigations into the molecular biology.

In this report, we briefly describe the response to ibrutinib in cases of MCL with CNS involvement as well as a recurrent observation, not previously reported to this extent. While cytogenetic changes are frequent in MCL both at diagnosis and relapse, with translocation t(11;14) being a prominent hallmark of the disease, somatic copy number alterations of chromosome 1q are, to our knowledge, very rare. Yet, we successively report four whole-exome sequenced patients, of which three evidently display large aberrations of chromosome 1, two being deletions and one a copy gain. Two of the patients progressed with 1q copy alterations at CNS relapse detected in cerebrospinal fluid (CSF), while another carried the deletion at diagnosis (Table 1). Patients 1, 3, and 4 were found to have acquired large 17p deletions by assessment of variant allele frequencies (VAF). These findings were accompanied by mutations in TP53 for patients 1 and 2 at diagnosis (Table 1 and 2).

**Table 1:** **Patient characteristics.** LDH: lactate dehydrogenase, U/L: units per liter, NA: not available, HDT+ASCT: High-dose therapy and autologous stem cell transplantation, CSF: cerebrospinal fluid, CNS: central nervous system, WES: analyzed with whole-exome sequencing, ~: approximately, *: SOX11 positivity was inconclusive at diagnosis but positive in the bone marrow two years later and prior to the first treatment regime, #: detected at diagnosis, $: detected at relapse. The chromosome 1q copy number alterations were found in CSF at relapse for patients 1 and 3, and in diagnostic lymph node for patient. 2 (sequencing of CSF at relapse failed with an exhaust of material).

Patient	1	2	3	4
Age at diagnosis (year)	72 (2015)	61 (2015)	62 (2014)	72 (2012)
Stage	IV B	IV B	IV A	IV A
LDH (U/L)	Elevated (228)	Elevated (244)	Normal (123)	Normal (158)
MIPI score	7,0	7,9	5,9	6,8
Ki67	45	90	NA	10
HDT+ASCT	–	+	+	–
Intracerebral lesion	+	–	+	–
Intraspinal lesion	–	+	+	–
Relapse CSF involvement	+	+	+	–
Relapse CNS involvement	+	+	+	+
Ibrutinib response duration at CNS relapse	<1 month	~1 month	~1 month	> 60 months
Status at last follow-up	Dead	Dead	Dead	Dead
Ibrutinib toxicity	–	Inversive aspergillus	–	Pleura effusion
CD5, CD19, CD20, CD22	+	+	+	+
CD10, CD23, CD200	–	–	–	–
SOX11	+	+	+*	+
CCND1	+	+	+	+
TP53 deletion (WES)	+^#$^	–	+^$^	+^$^
TP53 mutation (WES)	+^#^	+^#^	–	–
1q copy number alteration (WES)	+^$^	+^#^	+^$^	–

**Table 2:** **Detected somatic mutations.** Mutations overlapping with the Cancer Gene Census list provided by the Catalogue of Somatic Mutations in Cancer (COSMIC, v92, Tier 1 and 2) [11] are shown. The mutations were detected with Mutect2 (flagged PASS), with sequencing alignment in BWA (GRCh37) and processing in GATK 4.1.8 (Broad Institute, Cambridge, MA, USA) [10]. All mutations with a depth of coverage below 20 were discarded. Pt.: Patient number. DP: Depth of coverage. NA: Not available. ND: Not detected. *Relapse* denotes relapse *cerebrospinal fluid* for patients 1 and 3, *relapse bone marrow* for patient 2 with almost no detectable malignant cells (1%, see Fig. 1. Note that sequencing of relapse CSF failed for pt. 2), in agreement with mutational status, and relapse dorsal tumor for patient 4.

Pt.	Gene	Position	Identifier	Diagnosis DP (Lymph node)	Diagnosis (Bone marrow)	Control DP	Relapse DP
1	BCORL1	chrX:129,148,676 *C*>*A*		ND	ND	ND	6/31 (19%)
	CARD11	chr7:2,985,521 *A*>*T*	COSV62717189	50/193 (26%)	55/420 (13%)	ND	ND
	EBBR2	chr17:37,865,708 *A*>*G*	(splice junction)	26/53 (49%)	10/70 (14%)	ND	14/31 (45%)
	PBRM1	chr3:52,610,594 *A*>AC		33/93 (35%)	30/263 (11%)	ND	ND
	SMARCA4	chr19:11,123,693 *G*>*A*	COSV60798909	ND	14/129 (11%)	ND	14/46 (30%)
	TP53	chr17:7,577,515 *T*>*G*	COSV52801994	47/81 (58%)	37/198 (19%)	ND	ND
	UBR5	chr8:103,269,922 *G*>*A*		30/77 (39%)	44/231 (19%)	ND	43/73 (59%)
2	EGFR	chr7:55,087,012 *G*>*T*		56/154 (56%)	ND	ND	NA
	NUP98	chr11:3,793,053 *C*>*T*	rs148092095	185/370 (50%)	ND	ND	NA
	SF3B1	chr2:198,261,029 *A*>*T*		62/203 (31%)	5/193 (3%)	ND	NA
	TP53	chr17:7,577,541 *T*>*A*	COSV52732730	144/331 (44%)	4/212 (2%)	1/164 (1%)	NA
	TP53	chr17:7,578,546 AG>*A*		237/299 (79%)	5/323 (2%)	ND	NA
3	CCND1	chr11:69,457,900 *G*>*A*		NA	ND	ND	88/109 (81%)
	CSMD3	chr8:113,694,865 *G*>*A*		NA	22/153 (14%)	ND	50/117 (43%)
	NSD2	chr4:1,962,801 *G*>*A*	COSV56386422	NA	31/266 (12%)	1/567 (0%)	150/237 (63%)
	ROBO2	chr3:77,681,753 *G*>*C*		NA	22/269 (8%)	1/349 (0%)	50/156 (32%)
	SLC45A3	chr1:205,632,142 *C*>*A*		NA	8/29 (28%)	ND	ND
4	ATM	chr11:108,216,614 AG…>*A*		NA	27/81 (33%)	ND	27/89 (30%)
	DDX5	chr17:62,500,098 TACAG>*T*	rs782442161	NA	2/562 (0%)	ND	102/134 (76%)
	IRS4	X:107,977,919 *G*>*T*	rs199512071	NA	7/47 (15%)	ND	ND
	PTPRK	chr6:128,505,823 *T*>*C*	COSV100949489	NA	ND	ND	51/138 (37%)

## 2 Patient cases

All four patients had a consistent immunophenotype evaluated by flow cytometry (FC) from diagnosis to relapse, being positive for CD5, CD19, CD20, CD22, and either kappa or lambda expression, while showing CD10, CD23, and CD200 negativity (Table 1). Additional individual markers were employed in FC and immunohistochemistry staining for histopathological confirmation of MCL, and exclusion of CLL or other lymphomas. In the same manner, CCND1 and PAX5 expression was consistently evaluated at diagnosis, together with SOX11, and at relapse. All patients received rituximab as part of the first-line treatment regimens in combination with bendamustine (patient 1) and R-CHOP (patient 2, 3, 4), alternating with rituximab and high-dose cytarabine followed by autologous stem cell transplantation (patient 2, 3). All patients initially responded to ibrutinib treatment after CNS relapse, evaluated by the clinical response in three patients and by CNS cytoreduction in one patient who did not have clinical symptoms. Time to response varied from 4 days to 4 weeks.

*The level of details for each report is deliberately decreased, successively, due to space limitations and scope. The status of chromosome 1q aberrations and somatic mutations were unknown at the time of sampling, and the patient cases were selected and sequenced for this study solely on the basis of receiving ibrutinib following CNS relapse. Methods are found in the online supplement together with a complete list of somatic mutations.*

**Fig. 1:**
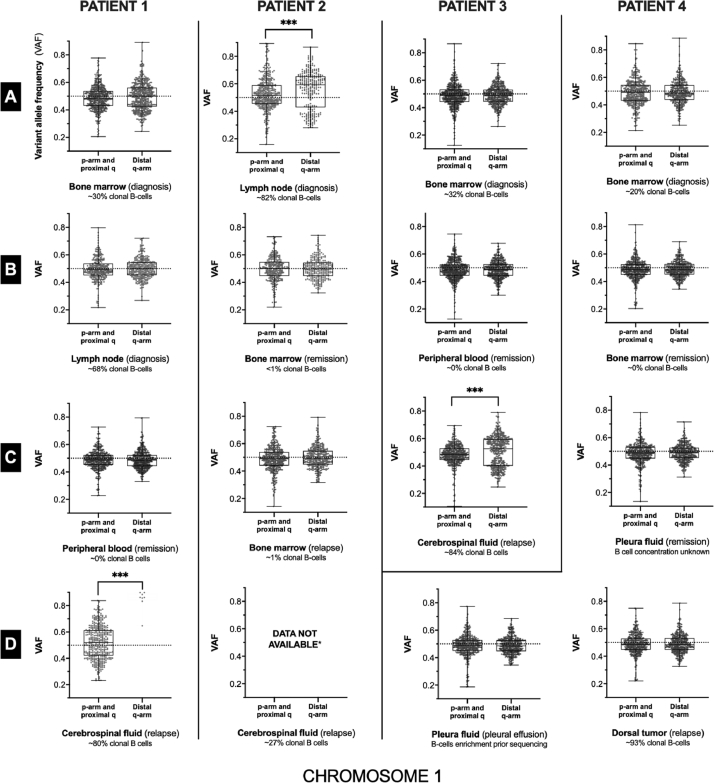
**Allelic imbalance of chromosome 1q was observed in three of the four mantle cell lymphoma cases: in two at relapse in the central nervous system (patient 1 and 3, cerebrospinal fluid (CSF)) and one at diagnosis (patient 2).** Each individual plot displays the allele frequencies of heterozygous variants (VAF) on chromosome 1 and significant deviation, or imbalance, from normal disomy. In all three cases, the aberration spanned almost 100 megabases distal to the centromere, and thus the majority of the q-arm. Sequencing of patient 2 CSF relapse material failed and thus could not be evaluated (*). All three evident allelic imbalances on the q-arm were significantly different from the p-arm (***, *Wilcoxon rank-sum test:p* ≤ 10^−4^, *Kolmogorov-Smirnov test of equal distributions: p* ≤ 10^−4^).

**Fig. 2:**
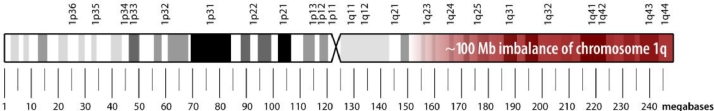
**The size of the allelic imbalance on chromosome 1q** was found to be approximately 100 megabases distal to the centromere across the three patients with apparent copy-alterations (the gradient marks the approximate starting point of the lesion of the four patients, as it is not possible to conclude the exact position from the whole-exome sequencing resolution).

*Patient 1:* Male, age 72 at MCL diagnosis ultimo 2015 (stage IV B, Ann Arbor classification, Table 1). Malignant B cells from the inguinal lymph node were assessed pleomorphic at diagnosis, staining positive for CCND1, SOX11, PAX5, and BCL2 by immunohistochemistry in addition to previously described surface markers, while being BCL6 negative. FC detected approximately 68% lymphoma cells in a lymph node and 30% in bone marrow (BM). Complete remission was attained following treatment with rituximab and bendamustine. Onset of nausea, visual disturbances, and aphasia occurred within a year of the diagnosis. Only cells with normal karyotype were found in BM at relapse, while CSF contained an excess of 300,000 B cells/ml with meningeal involvement demonstrated by cerebral magnetic resonance imaging. Treatment with ibrutinib and prednisolone was initiated resulting in reestablished vision, nonimpaired speech and movements after a week together with a marked decrease in the number of cells in spinal fluid to 85,000 cells/ml. Neurological symptoms reappeared after 4 weeks, and the patient progressed ad mortem 8 weeks after CNS relapse. A high burden deletion (~80%) was found at relapse on chromosome 1q (approximately 95 Mb, Fig. 1, patient 1 panel D, and Fig. 2) by whole-exome sequencing (WES). *UBR5* (8:103,269,922 *G*>*A*, nonsense mutation, GRCh37) and *ERBB2* (17:37,865,708 *A*>*G*, intron-exon splice junction) were shared between diagnostic lymph node, BM, and relapse CSF (Table 2). Both a TP53 mutation and 17p deletion were found by sequencing of diagnostic sample.

*Patient 2:* Woman, age 61, diagnosed following inguinal biopsy (stage IV B). The biopsy was positive for SOX11. The patient showed approximately 80% lymphocytes in BM by microscopic examination of imprint slides and was positive for PAX5 and CCND1 by immunohistochemistry. R-CHOP based immuno-chemotherapy was initiated and high-dose therapy and autologous stem cell transplantation (HDT+ASCT) was completed six months after diagnosis. CNS relapse occurred 10 months after diagnosis. CNS involvement was proven in CSF shortly after partial remission in BM (~1% residual MCL cells) with a concentration of well over a thousand cells per milliliter with approximately half of the leucocytes being lymphoma cells evaluated by FC. Ibrutinib treatment was initiated as second-line treatment, followed by immediate cytoreduction from 1200 cells/ml to 100 cells/ml sustained ad mortem. The patient succumbed during invasive aspergillosis one and a half month after CNS relapse. The B cell lymphoma displayed double mutated TP53 (Table 2), negative for 17p deletion. In addition, a distal 1q deletion of approximately 100 Mb was detected utilizing VAF and read depth ratio analyses of tumor-control pair (Fig. 1, patient 2, panel A), with an estimated cell fraction of 25–30%.

**Fig. 3:**
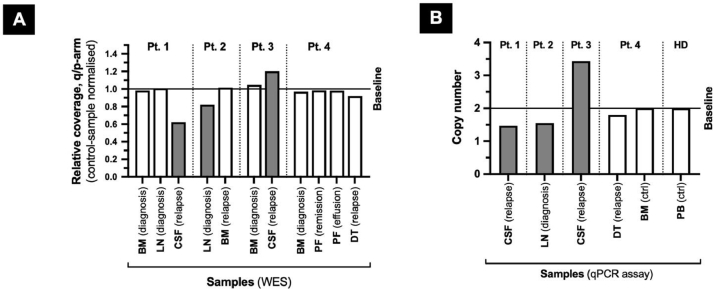
**Coverage of the chromosome 1 q-arm region afflicted by allelic imbalance relative to the unaffected p-arm.** Chromosome 1q of patient (pt.) 1 and 2 were evident of a copy-loss (A, dark gray), while a copy-gain was indicated for patient 3 (dark gray). All read depth ratios were normalized to that of the respective paired control. Samples without statistically significant allelic imbalance on the q-arm (white) were in close agreement with the paired control samples. BM: bone marrow, LN: lymph node, CSF: cerebrospinal fluid, PF: pleura fluid, DT: Dorsal tumor. The chromosome 1q imbalance observed in patient 1, 2 and, 3 were supported by qPCR (TaqMan Copy Number Assay) targeting the ABL2 gene (chromosome 1q25.2) and the reference gene TERT (chromosome 5p15.33).

*Patient 3:* Male, age 62, diagnosed MCL (stage IV A) with 32% lymphoma cells evaluated by FC. The patient was treated with R-CHOP after 2 years of “watch and wait” approach. HDT+ASCT was completed six months after the start of therapy. PAX5 and CCND1 positivity was confirmed, while SOX11 was inconclusive at diagnosis and confirmed positive two years later at treatment initiation. TP53 expression was absent, and the karyotype was evaluated as being normal at diagnosis. CNS relapse occurred two years after diagnosis with 89% lymphoma cells in CSF, dominated by CD19, CD20, and kappa light chain positive cells. Several mutations were found to be shared between diagnosis and CNS relapse (Table 2). This patient also displayed a large allelic imbalance (Fig. 1, patient 3, panel C) of 95–100 Mb at the same location as patients 1 and 2 (Fig. 2), however showing evidence of a chromosome copy-gain (Fig. 3, patient 3). The estimated burden from variant allele frequencies was 60%. Ibrutinib treatment was initiated shortly after CNS relapse with a reduction of CSF leukocytes from 170,000 to 39,000 cells/ml within three weeks. The patient progressed four weeks after relapse was identified and died five weeks after.

**Fig. 4:**
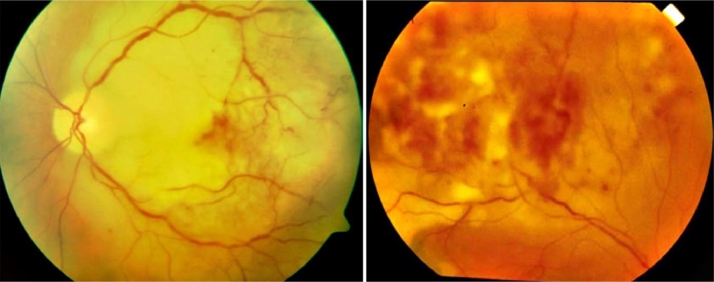
**Fundoscopy showing infiltration of malignant cells in corpus vitreum at relapse (left and right eye).** Patient 4 experienced a CNS relapse without cerebrospinal clonal B cells. The patient was still alive after 60 months, and the malignant cells did not show any aberrations on chromosome 1 in contrast to the other cases.

*Patient 4:* Male, age 72, diagnosed with MCL (stage IV A) followed by induction treatment with R-CHOP. Relapse occurred 2 years after diagnosis, where PET/CT showed bilateral intraocular tumor infiltration, and a dorsal tumor was detected with a high number of clonal B cells (93% by FC). Assessment of the infiltration of corpus vitreum by fundoscopy confirmed the CNS relapse (Fig. 4), while no malignant cells were present in the CSF. The patient responded immediately to ibrutinib treatment and attained complete remission, examined by PET/CT two months after onset. Despite recurrent pleura effusions, the patient remained in remission beyond 60 months. Measurable residual disease was below 0.04%, evaluated by FC, five years after diagnosis with an immunophenotype concordant with that of diagnosis. The most notable finding from WES was a double mutation in ATM, present at diagnosis and relapse, and 17p deletion (Table 2). *No copy number alteration on chromosome 1 was detected*.

## 3 Discussion

We have presented four cases of MCL, of which three were struck by an allelic imbalance/copy-alteration on chromosome 1q and displayed a very brief response to ibrutinib at CNS relapse. The treatment response was only sustained in one of the patients (pt. 4). This patient was devoid of malignant cells in CSF at CNS relapse and did not harbor a chromosome 1q loss or gain in the investigated materials. The WES copy-number profiling was based on the detection of allelic imbalances by variant allele frequencies and relative read-depth ratios, as previously implemented in the molecular profiling of four other patients with MCL using WES [6] (see also [7] and [5]), with the exception that the coverage of chromosome 1p arm was used for internal confirmation of the 1q copy number alteration, and to circumvent sequencing batch effects. All read depth ratios were normalized to that of the respective paired control.

While the knowledge supplements the current reports on MCL found within the CNS, by the fact that chromosome 1q copy number alterations are largely undescribed, *it remains unclear whether the lesions, comprising almost the entire chromosomal q-arm, contribute to any phenotypic attributes of CNS infiltrating MCL or whether these play any role in the treatment resistance to ibrutinib*. Chromosome 1p or 1q deletions and amplifications have been reported in other lymphomas [1, 4, 8] but extensive 1q copy-number alterations seem to be rare in MCL. A few cases of 1q deletions have previously been demonstrated in two MCL cell lines and five patient samples with single nucleotide polymorphism genomic microarrays [9], which are capable of finding both copy altering and copy-neutral events by allelic imbalance, but not to definitely deduce the copy state on its own. Such 1q aberrations are thus candidates for further investigations. We hope that this report may help other researchers to progress towards a better understanding of this often-aggressive disease.

## Data availability

Somatic mutation candidates are found in the online *supplement* (annotated Mutect 2 output, GATK 4.1.8.1)

## Ethical approval

The project was approved by the National Ethical Committee in Denmark (Approval no. 1,605,184).

## Author's contribution

All authors contributed to this work.

## Declaration of Competing Interest

The authors have nothing to disclose.

## Appendix Supplementary materials

Image, application 1

Image, application 2

Image, application 3

Image, application 4

Image, application 5

Image, application 6

Image, application 7

Image, application 8

Image, application 9

Image, application 10
